# Training Effects on ROS Production Determined by Electron Paramagnetic Resonance in Master Swimmers

**DOI:** 10.1155/2015/804794

**Published:** 2015-03-22

**Authors:** Simona Mrakic-Sposta, Maristella Gussoni, Simone Porcelli, Lorenzo Pugliese, Gaspare Pavei, Giuseppe Bellistri, Michela Montorsi, Philippe Tacchini, Alessandra Vezzoli

**Affiliations:** ^1^Istituto di Bioimmagini e di Fisiologia Molecolare, Consiglio Nazionale delle Ricerche, Via Fratelli Cervi 93, 20090 Segrate, Italy; ^2^Dipartimento di Fisiopatologia Medico-Chirurgica e dei Trapianti, Università di Milano, Via Fratelli Cervi 93, 20090 Segrate, Italy; ^3^Istituto per lo Studio delle Macromolecole, Consiglio Nazionale delle Ricerche, Via Bassini 15, 20133 Milano, Italy; ^4^Università Telematica S. Raffaele Roma, Via F. Daverio 7, 20122 Milano, Italy; ^5^EDEL Therapeutics S.A., PSE-B/EPFL, 1015 Lausanne, Switzerland

## Abstract

Acute exercise induces an increase in Reactive Oxygen Species (ROS) production dependent on exercise intensity with highest ROS amount generated by strenuous exercise. However, chronic repetition of exercise, that is, exercise training, may reduce exercise-induced oxidative stress. Aim of this study was to evaluate the effects of 6-weeks high-intensity discontinuous training (HIDT), characterized by repeated variations of intensity and changes of redox potential, on ROS production and antioxidant capacity in sixteen master swimmers. Time course changes of ROS generation were assessed by Electron Paramagnetic Resonance in capillary blood by a microinvasive approach. An incremental arm-ergometer exercise (IE) until exhaustion was carried out at both before (PRE) and after (POST) training (Trg) period. A significant (*P* < 0.01) increase of ROS production from REST to the END of IE in PRE Trg (2.82 ± 0.66 versus 3.28 ± 0.66 *µ*mol·min^−1^) was observed. HIDT increased peak oxygen consumption (36.1 ± 4.3 versus 40.6 ± 5.7 mL·kg^−1^·min^−1^ PRE and POST Trg, resp.) and the antioxidant capacity (+13%) while it significantly decreased the ROS production both at REST (−20%) and after IE (−25%). The observed link between ROS production, adaptive antioxidant defense mechanisms, and peak oxygen consumption provides new insight into the correlation between ROS response pathways and muscle metabolic function.

## 1. Introduction

Cells are exposed to a large variety of Reactive Oxygen Species (ROS) from both exogenous and endogenous sources. At appropriate concentration, ROS are known to act as important signaling molecules essential to cell function, playing various regulatory roles in cells [1]. Nevertheless the effects of ROS are dose dependent and when ROS generation exceeds antioxidant defenses oxidative damage is observed [2].

Exercise is associated with an increase in oxygen uptake by whole body and particularly by skeletal muscle [3], utilized, among others, into mitochondria for substrate metabolism and ATP production. As reported [4], an increase of 10-fold in the rate of whole body oxygen consumption and an increase of more than 100-fold in the oxygen flux in active muscles, during whole-body exercise, result in increased ROS formation, shifting the cellular environment from a reduced to an oxidized state, independently of physical activity types (aerobic, anaerobic, or resistance) [5]. Many factors might contribute to the oxidative stress induced by exercise also influencing the oxidative rate, such as recruited muscle groups, types of contraction, exercise frequency and intensity, and exercising population. Physical exercise is one of the most characteristic examples demonstrating that ROS are not necessarily harmful, considering that the well-known benefits of regular exercise on muscle function and health are accompanied by repeated episodes of oxidative stress [6]. The promoting effects of regular exercise on different cellular functions include the upregulation of antioxidant and oxidative damage repairing systems and induction of trophic factors [7]. Finally, training can play positive or negative effects on oxidative stress, depending on training load and specificity [8].

Previously it was demonstrated that high-intensity discontinuous and continuous moderate-intensity training induced similar beneficial effects in master runners, reducing the resting levels of oxidative stress biomarkers and inducing changes in total antioxidant capacity level [9].

Many investigators have assumed that skeletal muscle provides the major source of ROS generation during exercise [5]. Nevertheless, other tissues such as heart, lungs, or blood may also contribute to total body ROS generation during exercise [6]. Recent reports have indicated the potential role that blood may play at rest or during exercise on ROS production [10]. The whole blood or parts of it: plasma [11], erythrocytes [12], neutrophils [11, 13], lymphocytes [14], and platelets [15], have reported an increased production of various reactive species after exercise. However, the majority of the relevant human studies measured the redox status by using plasma. This probably can be ascribed to two reasons: (1) the assumption that plasma better reflects tissue redox status [16] and (2) the easiness of plasma collection. During exercise, ROS are generated by both blood and muscle and it is reasonable to assume that a corresponding systemic steady state level is reached in blood. The same may hold true for exchanges among blood constituents [6] once certain basic assumptions are met: reactive species with adequate half-life have the ability to cross membranes and generate new reactive species at the vicinity of the considered compartments.

Usually, direct measurements of free radical and reactive species production are very difficult due to their high reactivity and low steady-state concentration [17]. Consequently, for the assessment of oxidative stress, indirect methods are mainly used. Indeed, Electronic Paramagnetic Resonance (EPR) spectroscopy is the only technique available to directly detect the “instantaneous” presence and to quantitate ROS concentration in biological samples. Nevertheless ROS half-life (*t*_1/2_  (s): superoxide [O_2_^∙−^] 10^−4^; nitric oxide [NO^∙^] 4 · 10^−1^, at room temperature) is too short when compared to the EPR time scale so they are EPR invisible. This is only when “trapped” and transformed in a more stable radical species that they become EPR detectable. Moreover, in EPR spectra, signal areas are proportional to the number of the excited electron spins, leading to absolute concentration levels, when a stable radical compound is adopted as reference.

The present study aimed at examining the effects of high-intensity discontinuous training exercise on ROS production and on antioxidant capacity in master swimmers by applying reliable, rapid, and microinvasive EPR measurement of the instantaneous concentration of ROS [18, 19] and antioxidant power using a novel redox sensor to measure the levels of reducing species [20] directly in human peripheral blood. Possible correlation between metabolic and ROS production levels was also investigated.

## 2. Materials and Methods

### 2.1. Subjects

Sixteen master swimmers (males, mean age 30 ± 5 years; nonsmokers) of the Saronno Swimming Club were recruited. Athletes had a training experience of 11 ± 4 years and they were specialized in front crawl on distances between 50 and 400 m. All athletes belonged to the master swimmer category as established by both Féderation Internationale de Natation Amateur (FINA: http://www.fina.org/) (25-year-old subjects and over) and Italian Swimming Federation (FIN: http://www.federnuoto.it/). No special diet, minerals, vitamins, or other kinds of supplementation were administered to swimmers. During the experimental phase of the study antioxidant supply was excluded and participants sustained only the programmed training protocol. Furthermore, participants abstained from food (6 h) and physical activity, alcohol, and caffeine consumption (24 h) prior to testing and were not currently taking any medications or supplements. Subjects were tested after a week of tapering (PRE), characterized by low-intensity training of short duration.

The anthropometric characteristics and the calculated body mass index (BMI), body fat, and free fat masses, determined by bipolar bioimpedentiometry (Tanita), were assessed. A written informed consent was signed by all participants, after being informed of all risks, discomforts, and benefits associated with the study. Procedures were in accordance with the Declaration of Helsinki, and institutional review board approval was received for this study.

### 2.2. Experimental Protocol

All subjects visited the laboratory two times: before (PRE Trg (Trg = training)) and after (POST Trg) 6 weeks of high-intensity discontinuous training (HIDT). On the experimental day, the subjects arrived at the laboratory 2.5 h after consuming a standardized breakfast [77 percent energy (*E*%) carbohydrate; 11* E*% protein; 12* E*% fat].

All tests were performed under close medical supervision and subjects were continuously monitored by 12-lead electrocardiography (ECG). Participants sat on the arm crank ergometer (Monark 891E, Stockholm, Sweden) with the crankshaft in line with the shoulder joint [21]. The ergometer presented adjustable seat and handlebars, which were set to suit each subject. All subjects were instructed to remain seated during the test. Subjects performed an incremental exercise (IE) up to voluntary exhaustion. In brief, this protocol involved a starting power output of 15 W with increases of 15 W every 1 min up to voluntary exhaustion. Arm-ergometer workload was adjusted by manually placing weights on the attached basket. Cadence was set at 60 rpm. Pulmonary ventilation (*V*′*E*, expressed in BTPS), O_2_ uptake (*V*′O_2_), and CO_2_ output (*V*′CO_2_), both expressed in STPD, were determined breath-by-breath by a computerized metabolic cart (SensorMedics Vmax29c, Bilthoven, Netherlands). Expiratory flow measurements were performed by a mass flow sensor (hot wire anemometer), calibrated before each experiment by a 3-litre syringe, at three different flow rates. Tidal volume and *V*′*E* were calculated by integration of the flow tracings recorded at the mouth. *V*′O_2_ and *V*′CO_2_ were determined by continuously monitoring *P*O_2_ and *P*CO_2_ at the mouth throughout the respiratory cycle and from established mass balance equations, after alignment of the expiratory volume, expiratory gases tracings, and A/D conversion. Calibration of O_2_ and CO_2_ analysers was performed before each experiment by utilizing gas mixtures of known composition. Digital data were transmitted to a personal computer and stored on disk. Gas exchange ratio was calculated as *V*′CO_2_/*V*′O_2_. *V*′O_2_  peak was determined as the average of the last 20 s values. Heart rate (HR) was determined by ECG. Blood pressure (BP) was measured using a standard cuff sphygmomanometer. Severe hypertension (systolic BP value > 250 mmHg) or falling BP during exercise was adopted as criteria for terminating the test. At rest, at the end of exercise, and at 1, 3, and 5 min during the recovery period, blood lactate concentration ([La]_b_) was determined using an enzymatic method (Biosen 5030; EKF Diagnostic, Eppendorf, Milan, Italy) on 20 *μ*L of capillary blood obtained at the ear lobe.

Voluntary exhaustion was defined as the inability to maintain the armful frequency, despite vigorous encouragement by the operators, as well as by maximal levels of self-perceived exertion using the validated Borg scale [22].

### 2.3. EPR Measurements

**Figure 1:**
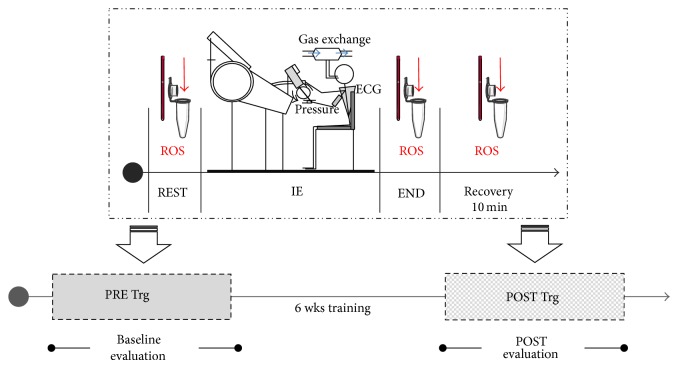
Sketch of the experimental protocol adopted to measure ROS production rate in swimmers. The data were collected at REST, at the END of the incremental arm-ergometer exercise (IE), carried out up to voluntary exhaustion, and at 10 min of the recovery period (see upper part of the figure) both before (PRE Trg) and after (POST Trg) training (lower part of the figure).

At rest, at the end of IE and after 10 minutes of recovery, ROS production rate was determined in 50 *μ*L capillary blood by means of a recently developed EPR microinvasive method [18, 19]. The capillary blood samples were collected at both PRE and POST Trg periods. The experimental protocol adopted for ROS detection in master swimmers is shown in Figure 1.

In summary, EPR experiments were carried out by using e-scan spectrometer (Bruker, Germany), operating at the common X-Band microwave frequency (~9.8 GHz). Acquisition EPR parameters were microwave frequency: 9.652 GHz; modulation frequency: 86 kHz; modulation amplitude: 2.28 G; sweep width: 60 G; microwave power: 21.90 mW; number of scans: 10; receiver gain: 3.17 · 10^1^. The instrument was interfaced to a temperature and gas controller unit (Bio III, Noxigen Science Transfer & Diagnostics GmbH, Germany) allowing temperature to be kept at the constant chosen level (37°C). Radical signals generated by the reaction of the 1-hydroxy-3-methoxycarbonyl-2,2,5,5-tetramethylpyrrolidine probe (CMH, Noxygen Science Transfer & Diagnostics, Germany) with the blood radicals were acquired and the spectra sequentially transformed for about 6 min in order to calculate the ROS production rate. The calculated spectral data were transformed in absolute concentration levels (*μ*mol·min^−1^) by recording the CP^•^ (3-carboxy-2,2,5,5-tetramethyl-1-pyrrolidinyloxy) stable radical signal adopted as reference (10 *µ*M). All EPR data were handled using the software standardly supplied by Bruker (Win-EPR version 2.11).

### 2.4. Antioxidant Capacity

Reducing capacity in blood was measured by a redox sensor in 10 *μ*L of capillary blood. The electrochemical measurements were performed using a commercial EDEL potentiostat electrochemical analyser (Edel Therapeutics, Switzerland) in a three-electrode arrangement. The working electrode (WE) was a screen-printed carbon electrode operating in conjunction with a screen-printed counter and a silver/silver-chloride (Ag/AgCl) reference one. This technique is an electrochemical-based method responding to all water-soluble compounds in biological fluids, which can be oxidized within a defined potential range [23, 24]. Blood sample was loaded onto a chip and an increasing potential between 0 and 1.2 V at a scan rate of 100 mV·s^−1^ (versus Ag/AgCl reference electrode) was applied while the resulting current was measured at the working electrode. The result was then pseudotitrated to account for the most biologically relevant antioxidants [20]. Data are expressed in nW.

### 2.5. Training Intervention

The training protocol adopted in our study, that is, HIDT, is characterized by brief intermittent bouts of vigorous activity, interspersed by periods of rest or low intensity exercise. HIDT causes repeated O_2_ consumption fluctuations related to changes of exercise intensity as opposed to continuous endurance training where O_2_ consumption is nearly constant during the exercise.

Subjects trained 3 times per week during 6 weeks in an indoor 25 m swimming pool. Training contents were classified in three intensity zones based on the individual anaerobic threshold (zone 1, 100–105% IAT; zone 2, 110–120% IAT; zone 3, >130% IAT). Total training volume and training amount at different intensity zones are presented in Table 1. The athletes' coach participated in the schedule of training programs and conducted all training sessions. Dry-land training (resistance, athletics, and cross training) was not performed.

At the start and end of each training session swimmers performed controlled warm-up (500 m per session) and cool-down (300 m per session), respectively. Excluding these phases in which subjects swam freely, all training sessions were conducted in front crawl.

### 2.6. Statistical Analysis

Descriptive statistics such as mean ± SD were used to summarize continuous variables. Data were analyzed using parametric statistics following mathematical confirmation of a normal distribution using Shapiro-Wilks W test. Experimental data were compared by ANOVA variance analysis followed by Bonferroni's multiple comparison test to further check among groups' significance (GraphPad Prism 6, Software Inc. San Diego, CA). The relationship between selected dependent variables was assessed using Pearson correlation coefficients. *P* < 0.05 statistical significance level was accepted.

Prospective calculation of power to determine subjects' number was made by using Freeware G^*^Power software (http://www.psycho.uni-duesseldorf.de/abteilungen/aap/gpower3/). At a power of 80% the number of subjects of 10 was calculated, which is well below the number of subjects recruited for this study.

## 3. Results

### 3.1. Exercise and Training Effects

Anthropometric characteristics and the main physiological variables recorded during arm cranking are reported in Table 2. After 6 wks of HIDT, *V*′O_2_ and power output peaks significantly (*P* < 0.001) increased in POST Trg versus PRE Trg.

**Figure 2:**
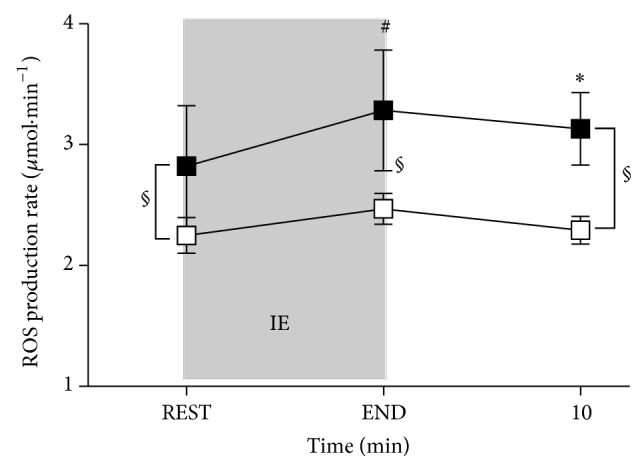
Time course of ROS production rate (*μ*mol·min^−1^) detected by EPR technique before (REST) and immediately after the IE (END) and at 10 minutes of recovery. The data obtained during two sessions of IE are shown: PRE Trg (full squares) and POST Trg (empty squares). Changes over time were significant at *P* < 0.05 during recovery (10 minutes after exercise) in PRE Trg (∗ symbol); *P* < 0.01 comparing peak levels in PRE Trg versus REST (# symbol); *P* < 0.001 between PRE Trg and POST Trg at REST, END, and 10 minutes of recovery (§ symbol).

The kinetics of ROS production data estimated by the EPR spectra recorded at rest, immediately after the IE, and at 10 min of recovery are shown in Figure 2.

After IE, ROS production increased significantly with respect to REST (*P* < 0.01) in PRE Trg (2.82 ± 0.66 versus 3.28 ± 0.66 *µ*mol·min^−1^) while the increase was not significant in POST Trg (2.24 ± 0.14 versus 2.46 ± 0.12 *µ*mol·min^−1^). Thereafter ROS production attained the resting levels in the time course of recovery, although in PRE Trg ROS level was found still more significantly (*P* < 0.05) higher (3.13 ± 0.30 *µ*mol·min^−1^) at 10 minutes of recovery in relation to REST.

**Figure 3:**
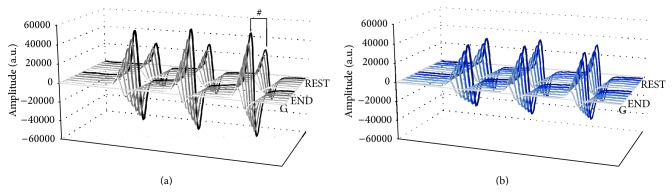
Stacked plots of the EPR spectra recorded at REST and at the END of exercise in PRE Trg (a) and in POST Trg (b). In each panel an increase of the signal amplitude (a. u.) at the end of exercise with respect to rest. A decrease of the signal amplitude in POST Trg with respect to PRE Trg shows up. # symbol (*P* < 0.01) shows the difference between REST versus END in PRE Trg.

HIDT induced a significant (*P* < 0.001) decrease in the ROS production rate at REST in POST Trg compared to PRE Trg (2.24 ± 0.14 versus 2.82 ± 0.66, resp.). Moreover, the attained peak value (END) resulted significantly (*P* < 0.001) lower in POST Trg than in PRE Trg despite a similar trend. Finally, a significant difference (*P* < 0.001) in the time course of recovery (10 minutes after exercise: 3.13 ± 0.30 versus 2.29 ± 0.11, resp.) between ROS production in PRE Trg and POST Trg was observed. Stacked plots of the EPR spectra recorded in PRE Trg at REST and at the END of exercise (a) and in POST Trg at REST and at the END of exercise (b) are shown in Figure 3. An increase of the signal amplitude (a. u). at the end of exercise with respect to rest and a decrease of the signal amplitude in POST Trg with respect to PRE show up from the spectra.

**Figure 4:**
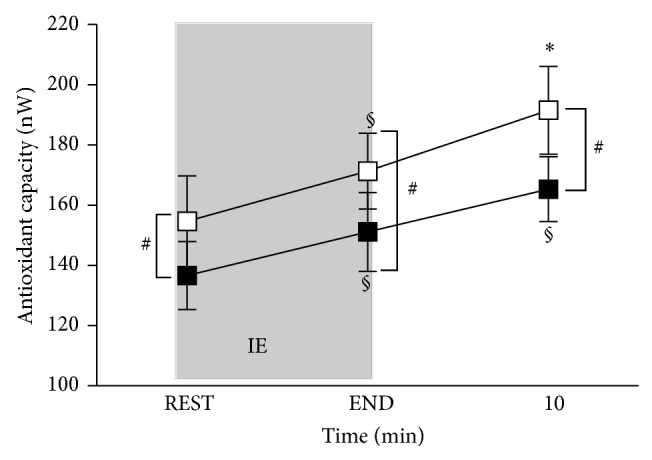
Time course of antioxidant capacity (nW) before (REST) and immediately after the IE (END) and at 10 minutes of recovery: PRE Trg (full squares) and POST Trg (empty squares). Changes over time were significant in PRE Trg at *P* < 0.001 at the END of exercise and during recovery (10 minutes after exercise) (§); in POST Trg at *P* < 0.001 at the END (§) and *P* < 0.05 during recovery (10 minutes after exercise) (∗); *P* < 0.01 between PRE Trg and POST Trg at REST, END, and 10 minutes of recovery (# symbol).

Antioxidant capacity changes after IE are displayed in Figure 4 as well. This parameter was found significantly increased with respect to the REST at the END and at 10 minutes of recovery, in both PRE (136.6 ± 11.34; 151.1 ± 13.1; 165.3 ± 10.9 nW, resp.) and POST Trg (154.7 ± 15.1; 171.4 ± 12.6; 191.5 ± 14.7 nW, resp.). HIDT induced a significant (*P* < 0.01) increase of antioxidant capacity in POST Trg compared to PRE Trg at REST and END and after 10 minutes of recovery (+13%; +13%; +16%, resp.).

**Figure 5:**
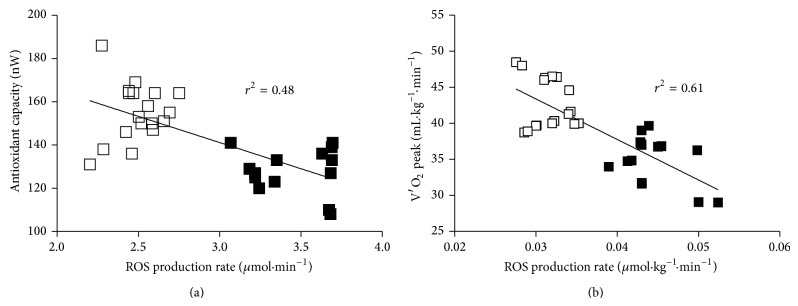
Panel plots of relationship between ROS production rate (*μ*mol·min^−1^) and (a) antioxidant capacity (nW) and (b) peak ROS production rate (*μ*mol·min^−1^·kg^−1^) and *V*′O_2_  peak (mL·kg^−1^·min^−1^) recorded at the end of IE in the two sessions: before (PRE Trg, full squares) and after (POST Trg, empty squares) training. The linear regression fit (solid line) is also shown and so is the correlation coefficient (*r* ^2^) reported in each panel. A significant linear relationship in the ROS production between antioxidant capacity (*P* < 0.0001) and *V*′O_2_ peak (*P* < 0.0001) values was estimated.

Lastly, a possible correlation between ROS production rate levels, antioxidant capacity, and metabolic data was investigated. An inverse significant relationship between (i) ROS production rate (*μ*mol·min^−1^) and antioxidant capacity (nW) (*r* ^2^ = 0.48, *P* < 0.0001) at baseline (see Figure 5(a)) and (ii) ROS peak production rate (*μ*mol·kg^−1^·min^−1^) and *V*′O_2_  peak (mL·kg^−1^·min^−1^) (*r* ^2^ = 0.61, *P* < 0.0001) (see Figure 5(b)) was found by Pearson's product-moment correlation.

## 4. Discussion

Many experimental works have analyzed the redox biology of exercise with high relevance to the area of Sport Science [17]: the general benefits of physical exercise are widely known and understood [25] but it is important to emphasize that exercise may generate an excessive production of free radicals [26]. As well-known and widely reported in the literature, compared to enzymatic methods able to measure end point biomarkers of oxidative stress damage (oxidized proteins and membrane lipids), EPR is the only technique allowing the direct detection and quantification of ROS. However despite the great interest in measuring ROS in biology and medicine, EPR technique has not till now been widely used because of several technical and methodological problems [27]. The observation that muscular exercise increases ROS production in skeletal muscles was for the first time reported by Davies et al. [28]. In the following years, a lot of studies on animals and humans have showed an increase of free radicals production after aerobic or anaerobic exercise in both sedentary or athletes subjects, according to exercise intensity [8, 29].

This increase was also observed in this study using an innovative method [18, 19] that employed EPR technique to attain a rapid and microinvasive measurement of ROS concentration in human peripheral blood. Compared with other spin trap and/or probe molecules, CMH was considered the spin probe of choice to quantify ROS in a most physiological way. Indeed, it shows greatest efficacy for trapping O_2_^∙−^ radicals, the reaction being much faster (1.2 × 10^4^ M^−1^ s^−1^) and producing stable CM-nitroxide, thereby enabling the reaction with both extra- and intracellular O_2_^∙−^. Moreover CMH detects ROS from all cellular compartments, including mitochondria [30].

During PRE and POST Trg sessions a significant increase of ROS production was found at the end of IE (+16% and +10%, resp.); this was followed by a gradual decrease in the magnitude of the ROS production in both sessions, returning toward resting values after 10 min (+11% and +2%, resp.). This finding is in agreement with the idea that increased ROS generation caused by physical exercise overwhelms the body capacity to detoxify ROS and that, upon chronic training, adaptive responses, including the one of the antioxidant defense system, better controls ROS production both at rest and after IE. Indeed antioxidant capacity significantly improved at REST (+13%) and maintained high levels 10 min after the end of the exercise (+16%).

One of the aims of this study was to investigate, by means of the same mini-invasive measurement method adopted for ROS production levels determination, whether alterations in redox homeostasis can be monitored to assess the fitness of intensively training athletes.

Aiming at minimizing the invasiveness of the method and hence to improve its potential for routine applications, oxidative stress markers (e.g., thiobarbituric acid substances, protein carbonyls) determination, requiring more invasive venous blood samples, was herein avoided. This choice was also supported by the linear correlation between ROS production rate and the above-mentioned biomarkers concentration previously observed at rest [18, 19]. In addition, the time-course changes of the same oxidative stress biomarkers were found delayed and of longer duration with respect to ROS production kinetics so that no correlation was possible in dynamic conditions [18].

The relationship between metabolic measurements and ROS production rate, before and after an exercise training program, was for the first time attempted in order to check the relation between antioxidant adaptive pathways and muscle metabolic function and at the same time investigate whether alterations in redox homeostasis can be monitored to assess the fitness of intensively training athletes. Indeed, in the examined athletes, exhibiting clear physiological training effects, a significant statistical inverse correlation was observed between ROS production rate and *V*′O_2_  peak (Figure 5(b)) determined during an IE. In support to our data, Bloomer et al. [31] demonstrated that peak protein carbonyl concentration value is a function of total *V*′O_2_.

It is known that *V*′O_2_ peak is one of the important parameters of physical fitness: thus an improvement of *V*′O_2_ after training accompanied with an increase in the antioxidant capacity and a subsequent decrease in ROS production can enhance the redox homeostasis thus producing beneficial effects on the response of human body to physical exercise. Moreover Venditti et al. [32] reported that chronic endurance training reduces H_2_O_2_ production from skeletal muscle mitochondria isolated from gastrocnemius muscles of Wistar rats, by reducing the production at complex I of the electron transport chain. Similarly in the present study ROS production lowered according to the training degree as seen in Figure 5(b) where subjects' fitness can be evaluated by the *V*′O_2_ peak values.

Finally the obtained results support that such HIDT protocol, characterized by repeated variations of intensity [33] associated with changes of redox potential, ATP/ADP ratio, and, consequently, disturbances of cellular homeostasis, can play a positive effect on oxidative stress leading to decrease in lipid peroxidation and DNA damage [9] and on antioxidant capacity reducing ROS production.

## 5. Conclusions

The study showed that 6 weeks of HIDT training improves exercise (+12% *V*′O_2_ peak) and antioxidant (+13%) capacity and significantly (*P* < 0.001) decreases baseline ROS production (−20%). Results also show that after identical exercise trained individuals produced lower levels of ROS related to higher level of antioxidant capacity compared to an untrained state. A novel insight into the correlation between ROS production response pathways and muscle metabolic function has been attained. The adopted microinvasive procedure for ROS rate production measurement by EPR appeared to be a reliable method to evaluate oxidative stress adaptation to acute exercise and training.

## Figures and Tables

**Table 1:** Weekly training contents were classified in three intensity zones based on the individual anaerobic threshold (IAT).

Training contents	HIDT	
	Distance (m)	% of total
Zone 1 (100–105% IAT)	600	10
Zone 2 (110–120% IAT)	2700	45
Zone 3 (>130% IAT)	2700	45

Total amount	6000	100

**Table 2:** Mean (±SD) values of the investigated variables obtained in the two sessions: PRE Trg (before training) and POST Trg (after training). BMI: body mass index; *V*′O_2_ peak: peak oxygen consumption; HR: heart rate; [La]_b_ peak: blood lactate peak concentration; BORG: scale measure of perceived exertion.

	PRE	POST
Weight (kg)	78.6 ± 5.0	78.8 ± 5.1
Height (cm)	182.2 ± 4.7	182.2 ± 4.7
BMI (kg·m^−2^)	23.7 ± 2.0	23.8 ± 2.0
Fat mass (kg)	13.3 ± 3.5	13.0 ± 2.9
Free fat mass (kg)	65.3 ± 2.3	65.7 ± 2.9
Peak power output (W)	175 ± 23	200 ± 26^#^
*V*′O_2_ peak (L·min^−1^)	2.87 ± 0.41	3.21 ± 0.56^#^
*V*′O_2_ peak (mL·Kg^−1^·min^−1^)	36.15 ± 4.26	40.64 ± 5.73^#^
HR peak (beats·min^−1^)	175.5 ± 5.38	174.90 ± 7.93
[La]_b_ peak (mM)	10.05 ± 2.00	11.85 ± 2.53
Borg scale	16.8 ± 1.68	17.2 ± 1.39

^#^Statistically significant difference at *P* < 0.01.
